# Reducing suicidal thoughts in the Australian general population through web-based self-help: study protocol for a randomized controlled trial

**DOI:** 10.1186/s13063-015-0589-1

**Published:** 2015-02-25

**Authors:** Bregje AJ van Spijker, Alison L Calear, Philip J Batterham, Andrew J Mackinnon, John A Gosling, Ad JFM Kerkhof, Daniela Solomon, Helen Christensen

**Affiliations:** National Institute for Mental Health Research, The Australian National University, Building 63, Canberra, ACT 2601 Australia; Black Dog Institute, The University of New South Wales, Hospital Road, Prince of Wales Hospital, Randwick, NSW 2031 Australia; Orygen Youth Health Research Centre, University of Melbourne, 35 Poplar Road, Parkville, VIC 3052 Australia; Department of Clinical Psychology and the EMGO Institute for Health and Care Research, Faculty of Psychology and Education, VU University Amsterdam, van der Boechorststraat 1, 1081 BT Amsterdam, the Netherlands

**Keywords:** Suicidal ideation, Suicidal thoughts, Internet, Web-based, Self-help, Cognitive behavioral therapy, Randomized controlled trial

## Abstract

**Background:**

Suicidal thoughts are common in the general population, causing significant disability. However, a substantial number of people struggling with suicidality do not access appropriate services. Online self-help may help overcome barriers to help-seeking. This study aims to examine the effectiveness of an online self-help program targeted at reducing suicidal thoughts compared with an attention-matched control condition in the Australian adult population. This trial is based on a Dutch self-help program, which was found to be effective in reducing suicidal thoughts.

**Methods/Design:**

A total of 570 community-dwelling adults (18 to 65 years old) with suicidal thoughts will be recruited via various media and randomly assigned to the 6-week online program aimed at reducing suicidal thoughts or a 6-week attention-matched control program. Primary outcome measure is the severity of suicidal thoughts. Secondary outcome measures include suicide plans, capacity to cope with suicidal thoughts, reasons for living, symptoms of depression, hopelessness, anxiety/worry, rumination, panic, perceived burdensomeness and thwarted belongingness, acquired capability, alcohol consumption, insomnia, and various cost-effectiveness measures.

**Discussion:**

Although the original Dutch trial found web-based self-help to be effective in reducing suicidal thoughts, randomized controlled trials (RCT) of online programs for suicidal thoughts are rare. The present study extends previous research by running the first English language RCT of this sort. As a result of the original study, the current RCT includes refinements to the design, including greater levels of participant anonymity and longer follow-up periods. Limitations of this trial include the potential for high drop-out and the inability to ascertain whether any suicides occur during the study.

**Trial registration:**

Australian New Zealand Clinical Trials Registry (ANZCTR) Registration number: ACTRN12613000410752 (15 April 2013). Universal Trial Number (UTN): U1111-1141-6595 (15 April 2013).

**Electronic supplementary material:**

The online version of this article (doi:10.1186/s13063-015-0589-1) contains supplementary material, which is available to authorized users.

## Background

Suicidal thoughts are common in the general population. A cross-national study of 17 countries reported an average lifetime prevalence of 9.2% for suicidal thoughts, 3.1% for plans, and 2.7% for attempts. However, there was considerable variability in prevalence rates between the countries [1]. In Australia, an estimated 13.3% of adults experience suicidal thoughts at some stage in their lives, 4.0% make a suicide plan, and approximately 3.2% of adults attempt suicide [2].

A significant proportion of people with suicidal thoughts or plans do not use services for mental health problems (in Australia, 40.9% and 32.0% respectively). In people who attempt suicide, about one in four (26.6%) fail to access services [2]. In a worldwide survey, it was found that 44% of people experiencing any suicidal outcome in high income countries do not receive treatment [3], indicating that current services do not reach everyone in need.

Reasons for a failure to seek help among suicidal people are diverse and likely to be complex. However, factors that may play a role include fear of stigma, shame, dissatisfaction or past negative experiences with mental health services, preference for self-reliance (handling the problem alone), denying the severity of the problem, and financial constraints [3-6].

It is widely acknowledged that suicide prevention services that are anonymous, confidential and readily available appeal to those at risk. Illustrative of this is the worldwide use of telephone support services. Although these are generally perceived as a valuable suicide prevention strategy, evidence for their effectiveness is limited and equivocal [7]. The internet is also increasingly being used as a source of information and of help and support for suicidal people and their loved ones, as demonstrated by the availability of peer-to-peer support forums, e-mail and chat therapy services, crisis chat services, and self-tests with advice and referrals [8-11]. Reviews of empirical studies into online suicide prevention efforts show that this is an emergent but promising field [12-14].

There is also recent evidence that web-based programs are associated with a reduction of suicidal thoughts. An uncontrolled study into web-based Cognitive Behavior Therapy (CBT) for depression found that suicidal ideation decreased post-treatment [15]. A randomized controlled (RCT) trial into online CBT for depression found reductions in suicidal thoughts irrespective of group allocation [16]. Both these earlier trials were of depression interventions. A Dutch trial studying an online unguided self-help program aimed specifically at reducing suicidal thoughts found this program to be effective compared to a control condition receiving psycho education [17,18]. To permit further and broader investigation, the Dutch program has now been translated into English.

This paper describes the protocol of the ‘Healthy Thinking’ trial, which aims to include 570 community dwelling adults living in Australia. Participants will be randomly allocated to one of the following conditions: ‘Living with Deadly Thoughts’ (LWDT) - the web-based self-help program aimed at helping people reduce suicidal thoughts, or ‘Living Well’ - an attention-matched control condition. This trial is based on the previous Dutch trial [19], but was adapted to the Australian context and improved. Limitations of the Dutch trial included the use of a waitlisted psycho-education control group, introducing unequal amounts of material presented to each group as a potential confound. The current trial utilizes an attention-matched comparator condition to account for this. In addition, participants in the Dutch trial were not anonymous, which may have impacted sample representativeness due to (un)willingness to participate. The current trial therefore offers anonymity as an option. Safety procedures developed specifically for the trial will operate while still permitting anonymous participation. These are documented here. Finally, the current trial employs a longer follow-up period to gauge longer-term maintenance of effects, and the look and feel of the program website has been improved to enhance user experience.

The primary objective of this parallel group RCT is to determine whether the Living with Deadly Thoughts program is superior to the Living Well program. It is hypothesized that participants receiving the LWDT program will have lower levels of suicidal thoughts on the Columbia-Suicide Severity Rating Scale (C-SSRS) relative to the control condition at post-test (6 weeks). The relative advantage of the active program compared to the control program is expected to be maintained at 6 and 12 month follow-up.

The key secondary outcomes include suicide plans, capacity to cope with suicidal thoughts, reasons for living, symptoms of depression, hopelessness, anxiety/worry, rumination, panic, perceived burdensomeness and thwarted belongingness, acquired capability, alcohol consumption, insomnia, help-seeking intentions and health care utilization. Relative to the control condition, LWDT participants are expected to 1) show a reduction of self-reported suicide plans at post-test and at 6 and 12 month follow-up; 2) show an improvement of capacity to cope with suicidal thoughts at post-test and 6 and 12 months; 3) experience an increase in ‘reasons for living’ at post-test, 6 and 12 months; 4) have lower symptoms of depression, hopelessness, anxiety/worry, rumination, panic, perceived burdensomeness, thwarted belongingness, and disability at post-test, 6 and 12 months; and 5) demonstrate an increase in help-seeking intentions and utilization of primary and mental health care at 6 and 12 months (but not necessarily in hospitalization).

For participants in the active group, improvements in suicidality (as measured by suicidal ideation, suicide plans and capacity to cope with suicide thoughts) are hypothesized to be associated with reduction of anxiety and depressive symptoms; a history of fewer self-reported attempts; shorter history of suicidal thinking; reduction in rumination; and improvement in perceived burdensomeness and thwarted belongingness. Improvement in suicidality is not expected to be related to changes in capability to attempt suicide, as per Joiner’s Interpersonal Theory of Suicide [20]. Stronger adherence to the program at 6 weeks is expected to be associated with lower baseline levels of hopelessness and higher baseline levels of internet literacy. Trial dropout at post-test, 6 and 12 months is hypothesized to be associated with higher baseline levels of hopelessness in both groups [18]. Other factors associated with adherence and drop-out will be explored.

## Methods/Design

### Design

**Figure 1:**
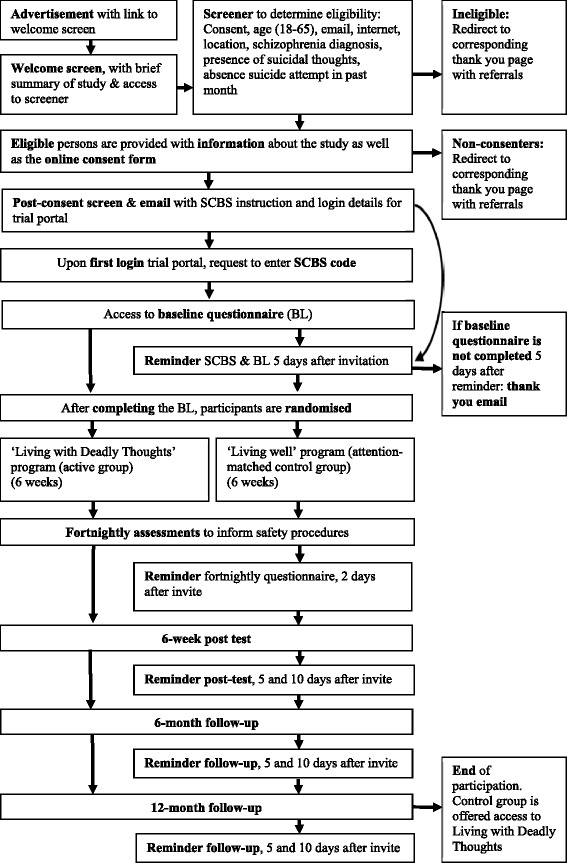
**Flow chart for the healthy thinking study.**

This study is designed as a two-arm randomized controlled trial with one active program (‘Living with Deadly Thoughts’) and one control program (‘Living Well’). There will be five main measurement occasions: screening, baseline, post-test (6 weeks after baseline), and two follow-up measures (6 and 12 months after post-test). In addition, suicidal thoughts will be monitored on a fortnightly basis during the first six weeks after randomization (that is, while the active group goes through the active program) to inform safety procedures (see below). The trial flow is illustrated in Figure 1. Participants who stop using the program they are assigned to will still be sent all follow-up questionnaires to complete (unless they have requested to be withdrawn from the study).

We adhere to the SPIRIT guidelines in reporting the trial in this protocol (see Table 1 for the items from the World Health Organization Trial Registration Data Set) [21,22].

**Table 1:** **Items from the World Health Organization Trial Registration Data Set**

**Data category**	**Information**
Primary registry and trial identifying number	Australian New Zealand Clinical Trials Registry: ACTRN12613000410752
Date of registration in primary registry	15 April 2013
Secondary identifying numbers	National Health and Medical Research Council project grant: GNT1046317
	Universal Trial Number: U1111-1141-6595
Source(s) of monetary or material support	National Health and Medical Research Council
Primary sponsor	
Secondary sponsor(s)	
Contact for public queries	B.A.J. van Spijker
Contact for scientific queries	B.A.J. van Spijker
Public title	Healthy Thinking project: A randomized controlled trial of a web-based self-help program to reduce suicide ideation
Scientific title	A randomized controlled trial comparing a web-based self-help program to reduce suicide ideation with an attention matched control program in community members with suicide ideation
Countries of recruitment	Australia
Health condition(s) or problem(s) studied	Suicidal thoughts
Intervention(s)	Active comparator: ‘Living with Deadly Thoughts’ program
	Control comparator: ‘Living Well’ program
Key inclusion and exclusion criteria	Ages eligible for study: 18 to 65 years; Sexes eligible for study: both; Accepts healthy volunteers: no
	Inclusion criteria: current suicidal thoughts
	Exclusion criteria: no current suicidal thoughts; suicide attempt in the past month; diagnosis of a psychotic disorder
Study type	Interventional
	Allocation: randomized; Intervention model: parallel assignment; Masking: participants blinded
	Primary purpose: prevention
Date of first enrollment	19 November 2013
Target sample size	570
Recruitment status	Recruiting
Primary outcome	Suicidal thoughts
Key secondary outcomes	Depression, hopelessness, reasons for living, anxiety, panic, alcohol use, insomnia, rumination, thwarted belongingness, perceived burdensomeness, acquired capability, health and disability, health care utilization, health status, help seeking, evaluation and utility of website

### Sample and recruitment

A total of 570 Australian community dwelling adults experiencing suicidal thoughts will be recruited through various online and offline media, relevant websites, popular social networking sites and advertising on popular search engines. Through these advertisements, respondents are linked to a welcome screen where a brief introduction to the study is provided. Next, respondents will be invited to provide informed consent to complete the online screening procedure to verify eligibility.

### Eligibility and screening

Eligibility is based on 1) being aged between 18 and 65, 2) having a valid email address, 3) having a reliable internet connection, 4) currently being located in Australia, 5) being fluent in English, 6) never having been diagnosed with a psychotic disorder such as schizophrenia, 7) currently experiencing suicidal thoughts, and 8) not having attempted suicide in the past month.

During screening, respondents indicate their eligibility by answering questions about the above criteria. Ineligibility during the screening process results in redirection to a ‘thank you’ page with relevant referral information. Respondents who are excluded based on a recent suicide attempt (past month) are also provided with the opportunity to submit their phone number to receive a phone call from the Suicide Call Back Service (SCBS: a 24-hour, Australia-wide, not-for-profit service that provides telephone counseling to people who are suicidal).

All eligible respondents are provided with a participant information statement, which outlines that participation includes agreeing to make contact with the SCBS and completing the baseline questionnaire before being randomly allocated to one of the two programs. Making initial contact with the SCBS is needed as this organization plays a significant role in the safety procedures (described in more detail below). After having read the participant information statement (see Additional file 1), respondents will be invited to provide informed consent online (see Additional file 2) and will need to provide only a valid email address and a first name to register their participation. They are also given the option to submit their phone number while consenting, which can then be used in the safety procedures if needed, but this is not mandatory for participation. This way, participants can remain anonymous if desired. See also Figure 1 for the trial flow-chart. Participants only enter the trial after they have provided informed consent.

### Random allocation procedure

Randomization to the active program or the comparator will be on a 1:1 ratio and based on blocks of length 4, stratified by sex and severity/extent of suicidal thoughts. The latter is defined as endorsing ‘yes’ on item 5 of the ‘Suicidal Ideation’ part of the Columbia-Suicide Severity Rating Scale (C-SSRS) [23], regarding active suicidal ideation with specific plan and intent. The randomization procedure is incorporated into the website and therefore fully automated. Participants are blinded as to which program is active and which is control. Participants in the control condition will receive access to the LWDT program after the 12-month follow-up assessment.

### Active condition: ‘Living with Deadly Thoughts’

#### Background

It has been convincingly demonstrated that online self-help interventions reduce mental health problems [24-29]. Self-help is nowadays often offered through the Internet and can be delivered with or without support. Guided self-help generally shows larger effect sizes [24] and lower dropout rates, whereas unguided self-help has the advantage that it can be delivered to a large number of people at relatively low cost.

There is a large body of research indicating that Cognitive Behavior Therapy (CBT) is effective for many different mental health problems [30], and most of the existing (effective) self-help interventions are based on CBT [31]. The program used in this trial is called Living with Deadly Thoughts and is a translation and adaptation of the Dutch web-based program ‘*Leven onder Controle*’ (literally ‘Living Under Control’). Its basis lies in evidence-based treatment programs for suicidality, mainly Cognitive Behavior Therapy (CBT); [32,33] and Dialectical Behavior Therapy (DBT); [34-37], but also Problem Solving Treatment (PST); [38,39] and Mindfulness Based Cognitive Therapy (MBCT); [40-42].

The main goal of the program is helping participants decrease the frequency and intensity of their suicidal thoughts. Special attention is given to the often repetitive character of suicidal thoughts [43]. As such, these thoughts are believed to be amenable to techniques found to be effective in reducing worry thoughts used in ‘Metacognitive Therapy for Worry and Generalised Anxiety Disorder’ [44]. From a patient perspective, these techniques are easy to grasp, offer novelty to those who have had prior CBT treatment, and can be readily taught as part of self-help care.

#### Structure

The program consists of six modules, and participants are encouraged to complete one module per week. Ideally, they spend 30 minutes per day on the program. Each module contains four components: 1) theory, 2) a weekly assignment, 3) two to three recommended exercises, and 4) several optional exercises to help consolidate relevant information and skills. In addition, at the end of each module a number of Frequently Asked Questions (FAQs) are listed that participants can refer to at any time. Each module builds on previous modules and these become available in sequence, 4 days after starting the previous module. Completed modules remain available throughout the intervention. Finally, participants also have access to referral information via a ‘get-help-now’ button that is visible from every page. Participants are also informed that participation is not meant to replace treatment as usual and are therefore encouraged to seek further treatment (if they are not receiving this already).

#### Content

In the first module, similarities between suicidal thinking and worry are outlined. Core exercises are aimed at recognizing how often suicidal thoughts are repeated and learning to manage this repetitiveness better. The second module focuses on learning to tolerate and regulate intense emotions. The theory section explains how to recognize an upcoming crisis and taps into dealing with the urge to self-harm. Core exercises introduce different ways of coping with intense emotions, such as behavioral activation (for example, seeking distraction), and acceptance (waiting until the feelings subside). Participants are also encouraged to make a crisis plan. The third, fourth, and fifth modules deal with identifying automatic thoughts, recognizing common thinking patterns (for example, all or nothing thinking, overgeneralization, and mind reading), and cognitively restructuring the three most important identified negative automatic thoughts. The final module is dedicated to relapse prevention. It discusses the possibility of future setbacks and disappointments. Core exercises pertain to envisaging a realistic picture of the future, dealing with future setbacks, and recognizing and preventing relapse by making a relapse prevention plan.

### Attention-matched control condition: ‘Living Well’

The attention-matched control condition uses a 6-week program that contains lifestyle information. This program follows a similar structure to the active program; that is, each module contains theory, a weekly assignment, several exercises, and a number of FAQs. Each module has a different topic: 1) nutrition, 2) a healthy home environment, 3) a healthy weight, 4) a healthy heart, 5) a healthy skin, and 6) a healthy mouth. The program was designed so that no specific mental health information or suicide-related content was included. New modules become available 4 days after starting the previous module. Similar to the active program, participants in the Living Well program have access to referral information via a ‘get-help-now’ button that is visible from every page and are informed that participation is not meant to replace treatment they may already be receiving or the necessity to seek further treatment.

### Measures

#### Primary outcome measure

The primary outcome measure is severity of suicidal thoughts, assessed using the ‘intensity of suicidal ideation’ section of the C-SSRS [23]. The C-SSRS was developed to identify those at risk and track changes in a person’s suicidal thinking and behavior over time. The full scale addresses the complete range of suicidal thinking and behavior and consists of three parts: 1) suicidal ideation, 2) intensity of suicidal ideation, and 3) suicidal behavior. Table 2 provides an overview of all measures used in the study.

**Table 2:** **Scales administered per measurement occasion in the healthy thinking study**

**Measure**	**Baseline**	**2 and 4 weeks**	**Post-test**	**6-month follow-up**	**12-month follow-up**
Demographics	X				
C-SSRS	X	X (3 items)	X	X	X
SIDAS	X		X	X	X
Brief COPE	X		X	X	X
BRFL	X		X	X	X
CES-D	X		X	X	X
Hopelessness scale	X		X	X	X
GAD-7	X		X	X	X
RRS	X		X	X	X
Brief PHQ (panic subscale)	X		X	X	X
INQ	X		X	X	X
ACSS	X		X	X	X
AUDIT-C	X		X	X	X
ISI	X		X	X	X
IEUQ			X		
GHSQ	X				
CSRI	X		X	X	X
SF-12	X		X	X	X
WHODAS 2.0	X		X	X	X

C-SSRS, Columbia Suicide Severity Rating Scale; SIDAS, Suicidal Ideation Attributes Scale; Brief COPE, Brief Coping Orientation Problems Experienced; BRFL, Brief Reasons for Living Scale; CES-D, Center for Epidemiological Studies - Depression; GAD-7, Generalized Anxiety Disorder-7; RRS, Ruminative Response Scale; Brief PHQ, Brief Patient Health Questionnaire; INQ, Interpersonal Needs Questionnaire; ACSS, Acquired Capability for Suicide Scale; AUDIT-C, Alcohol Use Disorders Identification Test Consumption; ISI, Insomnia Severity Index; IEUQ, Internet Evaluation and Utility Questionnaire; GHSQ, General Help-Seeking Questionnaires; CSRI, Client Service Receipt Inventory; SF-12, Short Form-12; WHODAS 2.0, World Health Organization Disability Assessment Schedule 2.0.

#### Secondary outcome measures

Secondary outcome measures include the following: reporting of suicide plans, measured by the ‘suicidal ideation’ section of the C-SSRS; capacity to cope with suicidal thoughts, using selected scales (self-distraction, active coping, positive reframing, planning, and self-blame) from the Brief Coping Orientation Problems Experienced (Brief COPE) [45]; reasons for living, using the Brief Reasons for Living Scale (BRFL) [46]; symptoms of depression, measured by Centre for Epidemiological Studies - Depression (CES-D) [47]; hopelessness, assessed by the Burns Hopelessness Scale (unpublished scale, Burns, D.D.); anxiety/worry, measured by the Generalized Anxiety Disorder-7 (GAD-7) [48]; rumination, assessed by the Ruminative Response Scale (RSS) [49,50]; panic, using the panic syndrome subscale of the Brief Patient Health Questionnaire (Brief PHQ) [51,52]; perceived burdensomeness and thwarted belongingness, assessed by the Interpersonal Needs Questionnaire (INQ) [53]; acquired capability, measured by the Acquired Capability for Suicide Scale (ACSS) [53]; alcohol consumption, using the Alcohol Use Disorders Identification Test Consumption (AUDIT-C) [54]; and insomnia, assessed by the Insomnia Severity Index (ISI) [55,56].

In addition, the following measures will be included to assess predictors of outcome and potential mediators of the effectiveness of the program: standard demographic information including internet literacy; history of suicidal thoughts, measured by number of months having experienced suicidal thoughts; history of suicide attempts, assessed by self-reported lifetime suicide attempts; adherence to the program, measured by website usage; evaluation of the program, using the Internet Evaluation and Utility Questionnaire (IEUQ) [57,58] and several additional questions; and help-seeking for suicidal thoughts, assessed by the General Help-Seeking Questionnaires (GHSQ) [59].

Finally, a recently developed scale, the Suicidal Ideation Attributes Scale (SIDAS) [60], is included in the study to further validate it in a help-seeking population and determine sensitivity to change. See also Table 1.

#### Cost-effectiveness

In addition to the outcomes described above, several measures are included in the study to allow for cost-effectiveness analyses. Specifically, the Client Service Receipt Inventory (CSRI) is used to collect data on health care utilization [61]. The CSRI can be tailored to suit data requirements, and in the current study the following domains will be captured: GP consultations, practice nurse visits, use of hospital services for physical problems, use of hospital services for mental health problems, mental health helpline contacts, psychiatric crisis support team contacts, social worker contacts, counseling contacts, therapy contacts, self-help group contacts, and psychiatrist contacts. In addition, the Short Form-12 (SF-12) [62] is included as a generic measure of perceived health status that can be used to estimate a preference-based single index for use in economic evaluations [63]. Similarly, the World Health Organization Disability Assessment Schedule 2.0 (WHODAS 2.0) is administered to assess generic health and disability [64,65]. Finally, costs related to the program are documented. See also Table 1.

### Safety of participants

As this research is being conducted in a vulnerable, at-risk population, safety procedures were designed in collaboration with the Suicide Call Back Service (SCBS), which is an Australia-wide 24-hour service that provides telephone and online counseling to people 15 years and over who are suicidal, are bereaved by suicide, have a suicidal loved one, or are professionals working with suicidal clients. The goal was to design a procedure that would protect and assist participants while enabling them to maintain anonymity within the trial if they chose.

To be able to enroll in the study, eligible participants will be required to contact the SCBS to obtain a unique code. They will be required to submit this code on their first login to the trial portal, before the participant is directed to the baseline questionnaire. This code subsequently enables researchers to identify the participant to the SCBS when needed (and vice versa), while not revealing the identity of the individual.

On each measurement occasion (that is, 2 and 4 weeks into the program, the week 6 post-test, 6-month follow-up and 12-month follow-up), the first three items of the ‘intensity of ideation’ section of the C-SSRS are used to detect high risk, defined as a score of 5 on any of these items. Whenever a participant meets this criterion, an automated email will prompt them to contact the SCBS. If they do not contact the SCBS within 2 days, an automated reminder will be sent. At the same time of this reminder, an automated email will be sent to the SCBS, which will contain the participant’s contact details if these were provided at registration. If no contact details were provided, the SCBS will take no further action; otherwise the SCBS will try to call the participant with a maximum of three attempts.

These safety procedures were designed from the perspective that participants should be able to maintain their anonymity as much as possible considering ethical and clinical obligations and also should be allowed to remain autonomous in their decision to make contact with services as much as possible. This approach is consistent with the concept of self-help and patient empowerment.

### Sample size and power

Power to detect change in suicidal thoughts is based on the expected effect of the intervention on the primary outcome measure. Based on the findings from the Dutch trial [18], the expected effect size is 0.30 (Cohen’s d). To be able to detect this effect size, assuming a correlation between pre- and post-test measures of .5, including an expected drop-out rate of 30%, and allowing a width of about ± 0.20 for the confidence intervals of the effect size, 285 starters (expected to yield 200 completers) would be needed in each condition (with alpha = 0.05 and power of 0.80). The aim is therefore to recruit 570 participants in the trial.

### Statistical considerations

Primary analyses will be undertaken on an intention-to-treat basis, including all participants randomized regardless of their level of adherence to the program or trial drop-out (unless someone specifically requests to remove their data from the study). Available information from all participants will be retained using linear mixed models analyses. Transformation of variables will be done as necessary to meet distributional assumptions and to accommodate potential outlying observations. For other analyses, multiple imputation including demographic and other background variables will be used as necessary to allow inclusion of data from all participants.

### Data Safety Monitoring Board

A Data Safety Monitoring Board (DSMB) has been established for this study, consisting of two senior psychiatrists and one senior suicidologist. All members have extensive clinical and/or research experience with suicidal people. This DSMB is independent of all investigators and the funding agency, and no member of the DSMB has direct involvement in the conduct of the study. The DSMB will receive monthly reports from the trial manager detailing the number of participants contacting the SCBS and number of self-reported suicide attempts on completed follow-up questionnaires. Furthermore, the DSMB will be notified of any adverse events within 72 hours of knowledge of the event. The DSMB will also receive a report if there is any evidence suggesting potential risk to participants, for example, as detected by the trial manager or chief investigators. Finally, interim analyses will be performed once one-third of the total sample (that is, 190 participants) has reached 1) the 6-week post-test assessment, 2) the 6-month follow-up, and 3) the 12-month follow-up. The DSMB may recommend termination or modification of the study on the basis of any perceived safety concern based on their clinical judgment. This may include, but is not limited to, higher anticipated rates of primary safety indicators (suicidal ideation and attempted suicide, both assessed by self-report), or unexpected serious adverse events.

### Ethics approval, data storage, and dissemination of results

This study (protocol version 2, 17 June 2013) has been approved by the Human Research Ethics Committees of the University of New South Wales (#HC13117) and the Australian National University (2013/416). Any modifications to the protocol will be agreed upon by the researchers and approved by the ethics committees prior to implementation. Participant responses will be identified by a unique user ID. Data files linking email addresses to ID numbers will be stored separately from raw research data. Collected data will be stored on a secure server. All chief investigators will be given access to the data set, and data will be published in nonidentifiable form. Authorship will be based on contribution to the study. A de-identified data set will be available from the authors no later than 3 years after collection of the final post-test data.

## Discussion

This paper describes the study protocol of a randomized controlled trial comparing a web-based self-help program for suicidal thoughts with an attention-matched program. This trial is a continuation of the first study that examined the effectiveness of web-based self-help for suicidal thoughts in the Netherlands [17-19] and is the first English-language RCT of this sort. The use of an attention-matched comparator condition, increased levels of participant anonymity, longer follow-up periods, and an improved look and feel of the program website improved the design of the study.

This study will contribute to the growing body of evidence regarding the effectiveness of online suicide prevention. Currently, there is evidence indicating that a reduction in suicidal thoughts is associated with the use of web-interventions [15,16], and that the effect is greater than waitlist control condition [18]; but more research is needed to establish that programs targeting suicidal thoughts are more effective than wait lists. If effective, web-based self-help has the potential to be implemented and disseminated in a cost-effective way [17,66-68].

Limitations of this study include the potential for high drop-out, to which web-based self-help programs are often subject [69,70] and may introduce selection bias. The anonymity of the participants will make it impossible to ascertain whether any suicides occur during the study. Similarly, the recording of suicide attempts relies solely on self-report and cannot be verified by objective records. Both these limitations are inherent to the self-help character of the trial and mimic real-life conditions if the program were to be implemented after the trial.

## Trial status

Recruitment started in November 2013 and is due to finish in 2015. The final participants are expected to complete their 12 month follow-up assessment in 2016.

## Additional files

Additional file 1:
**Participant information statement.**
Additional file 2:
**Consent form.**

## Abbreviations

ACSS: Acquired Capability for Suicide Scale
AUDIT-C: Alcohol Use Disorders Identification Test Consumption
Brief COPE: Brief Coping Orientation Problems Experienced
Brief PHQ: Brief Patient Health Questionnaire
BRFL: Brief Reasons for Living Scale
CBT: Cognitive Behavior Therapy
CES-D: Center for Epidemiological Studies - Depression
CSRI: Client Service Receipt Inventory
C-SSRS: Columbia-Suicide Severity Rating Scale
DBT: Dialectical Behavior Therapy
DSMB: Data Safety Monitoring Board
FAQs: frequently asked questions
GAD-7: Generalized Anxiety Disorder-7
GHSQ: General Help-Seeking Questionnaire
IEUQ: Internet Evaluation and Utility Questionnaire
INQ: Interpersonal Needs Questionnaire
ISI: Insomnia Severity Index
LWDT: Living with Deadly Thoughts
MBCT: Mindfulness Based Cognitive Therapy
PST: Problem Solving Treatment
RCT: randomized controlled trial
RRS: Ruminative Response Scale
SCBS: Suicide Call Back Service
SF-12: Short Form-12
SIDAS: Suicidal Ideation Attributes Scale
WHODAS 2.0: World Health Organization Disability Assessment Schedule 2.0
