# Supplementary material for: Reducing suicidal thoughts in the Australian general population through web-based self-help: study protocol for a randomized controlled trial
Source: Trials. 2015 Feb 25;16:62. doi: 10.1186/s13063-015-0589-1 (PMC4348119; doi:10.1186/s13063-015-0589-1)
Supplement: Additional file 1: — Participant information statement. [file 13063_2015_589_MOESM1_ESM.pdf]

Approval No 13117

PARTICIPANT INFORMATION STATEMENT

‘Healthy Thinking’ research study

You are eligible for participation in our study of web-based programs for suicidal thoughts and we would like to give you more detailed information about the study (see below) before you decide to participate. Please read the information carefully before clicking ‘next’ to go to the consent form.

Purpose of study

By conducting this study, we hope to learn whether web-based self-help can help reduce suicidal thoughts. We are looking to include 570 participants in the trial, who are all recruited via advertisements on the Internet and in the media.

Before you enter the study

Because we want to make sure that you are as safe as possible during the study, we ask that you register with the Suicide Call Back Service before you enter the study. The Suicide Call Back Service is an independent, not for profit, non-government organisation that aims to develop wellbeing and supports people to manage crisis through telephone based services. Registering with them involves making a phone call to tell them that you are participating in this study, after which they will provide you with a unique code. We will link this code to an ID number we assign to you. This way, you will not need to provide us with identifying information. However, you will be given the option to provide identifying information (phone number) to the Suicide Call Back Service if you want, but this is not obligatory for your participation in this study. Similarly, you will be given the option to enter your phone number on our website when you give your consent for the study, but this is not mandatory for participation. If during the study we have any indication that you may not be as safe as we would like you to be, you will receive an e-mail from us asking you to call the Suicide Call Back Service to chat about how you’re going. If you provide your phone number, the Suicide Call Back Service could use this to contact you if needed. After having consented to participate in the study, you will be provided with information on how to obtain the code from the Suicide Call Back Service, which you will then need to finalise your enrolment in the study.

Description of the study and the risks

If you decide to participate, we will provide you with secure access to the ***Healthy Thinking*** online trial portal, where you will be asked to enter your Suicide Call Back Service code and fill out a questionnaire (known as a pre-assessment or baseline questionnaire). This will take you approximately 30 minutes.

Once you have filled this out, you will be randomly (that is: by chance – like rolling a die or tossing a coin) assigned to one of two programs. In the first, you will be asked to monitor your suicidal thoughts and then to undertake training in how to control your suicidal thoughts. In the second, you will regularly be asked questions about your suicidal thoughts, and you will be asked to read information about how to improve your general health. Both programs are designed to be worked through independently, which means that no guidance is provided. Both programs will take 6 weeks to complete and optimally will require approximately 15-30 minutes per day of your time to read and practice. You will be able to start the program you are assigned to straight away.

While doing the program, you will receive a questionnaire about your suicidal thoughts every other week (i.e. in week 2 and week 4), which will take no more than 5 minutes to complete. Also, we may send you reminders to continue the program if we have indications that you have stopped using it. Six weeks after filling in the pre-assessment, we will ask you to repeat this online questionnaire. This is called the post-assessment and will tell us whether the programs have had an effect. Similarly, you will receive invitations to complete questionnaires 6 and 12 months after you finished the program. These so called 'follow-up assessments' will tell us whether effects are maintained after these periods.

We believe that most people participating in the study will find it valuable. There are no physical risks of being in this study other than those associated with suicidal thoughts. However, this study may require that you keep track of your suicidal thoughts, which can make you feel like you're thinking more about it at first. As common sense would suggest, we do not recommend that you continue the program if you feel that your suicidal thoughts are increasing substantially. At all times, we want you to be safe and we will do our best to help you achieve this. In addition, some people may not like completing the online questionnaires, in which case we also recommend that you stop.

Participation in this study may help you gain more control over your suicidal thoughts, and therefore reduce the number of times you have them and / or decrease their intensity. However, we cannot guarantee or promise that participating in this study will help you, because we need to do the research first to see if the programs work.

If you already are receiving help for your suicidal thoughts, or want to seek help during the study, this will not affect your possibility to (continue to) participate in the study. We do however advise you to inform your mental health care provider of your participation, but in the end it's up to you to decide whether you do.

Finally, we would like to advise you that the programs offered in this study are not a substitute for regular face-to-face help. However, we understand that it can be difficult to take the step to seek help, and that's why we provide this program. In keeping with the nature of the program, the only contact with our research team during the study period will be via the Internet.

Confidentiality and disclosure of information

Any information that is obtained in connection with this study and that can be identified with you will remain confidential and will be disclosed only with your permission, except as required by law. If you give us your permission by ticking 'yes' on the consent page, we plan to use the results to

determine whether the program has had an effect. Also, results may be discussed / published in scientific meetings or journals. In any publication, information will be provided in such a way that you cannot be identified.

Complaints

Complaints may be directed to the Ethics Secretariat, The University of New South Wales, Sydney 2052 Australia (phone 9385 4234, fax 9385 6648, email ethics.sec@unsw.edu.au). Any complaint you make will be investigated promptly and you will be informed of the outcome.

Feedback to participants

If you wish to be informed of the results of the study, you will be given the opportunity to sign up for a newsletter, which will be sent out at the completion of the trial (expected in 2016).

Your consent

Your decision whether or not to participate will not prejudice your future relations with the Black Dog Institute, the University of New South Wales or the Australian National University. If you decide to participate, you are free to withdraw your consent and to discontinue participation at any time without prejudice.

If you have any questions, please feel free to ask us via email:
healthythinking@blackdogstudies.org or call Dr Daniela Solomon (02 9382 9274).

If you decide to participate and give your consent (on the next page), you will be emailed a copy of this information sheet and your consent form to keep.
