# Supplementary material for: Reducing suicidal thoughts in the Australian general population through web-based self-help: study protocol for a randomized controlled trial
Source: Trials. 2015 Feb 25;16:62. doi: 10.1186/s13063-015-0589-1 (PMC4348119; doi:10.1186/s13063-015-0589-1)
Supplement: Additional file 2: — Consent form. [file 13063_2015_589_MOESM2_ESM.pdf]

Approval No 13117

CONSENT FORM

'Healthy Thinking' Study

You are making a decision whether or not to participate. Please note that consenting to participate implies that you understand that:

- Participation includes making contact with the Suicide Call Back Service.
- You will be sent questionnaires to fill in and possibly reminders to do so.
- You will be randomly assigned to one of two programs.
- Both these programs are unguided and therefore not monitored.
- We want you to be safe during the study and may therefore encourage you to contact the Suicide Call Back Service if needed.
- You will be given the option to provide your phone number, which can then be used by the Suicide Call Back Service to contact you if needed.

I have read the information provided and have decided to participate in this study.

- ○ YES
- ○ NO

Submit

(When yes is ticked, people are asked to fill in their e-mail address and first name. They are also given the option to fill in their phone number).
